# A new scale to assess health-facility level management: the development and validation of the facility management scale in Ghana, Uganda, and Malawi

**DOI:** 10.1186/s12913-024-10781-y

**Published:** 2024-03-25

**Authors:** Paul Mubiri, Freddie Ssengooba, Thomasena O’Byrne, Adelaine Aryaija-Keremani, Justine Namakula, Kingsley Chikaphupha, Moses Aikins, Tim Martineau, Frédérique Vallières

**Affiliations:** 1 School of Public Health, Makerere University, Kampala, Uganda; 2 Trinity Centre for Global Health, Trinity College Dublin, 7-9 Leinster Street South, Dublin 2, Dublin, Ireland; 3 Liverpool School of Tropical Medicine, Liverpool, UK; 4 Research for Equity and Community Health (REACH) Trust, Lilongwe, Malawi; 5 School of Public Health, College of Health Sciences, University of Ghana, Accra, Ghana

**Keywords:** Leadership, Management, Health facility

## Abstract

**Background:**

The increased recognition of governance, leadership, and management as determinants of health system performance has prompted calls for research focusing on the nature, quality, and measurement of this key health system building block. In low- or middle-income contexts (LMIC), where facility-level management and performance remain a challenge, valid tools to measure management have the potential to boost performance and accelerate improvements. We, therefore, sought to develop a Facility-level Management Scale (FMS) and test its reliability in the psychometric properties in three African contexts.

**Methods:**

The FMS was administered to 881 health workers in; Ghana (*n* = 287; 32.6%), Malawi (*n* = 66; 7.5%) and Uganda (*n* = 528; 59.9%). Half of the sample data was randomly subjected to exploratory factor analysis (EFA) and Monte Carlo Parallel Component Analysis to explore the FMS’ latent structure. The construct validity of this structure was then tested on the remaining half of the sample using confirmatory factor analysis (CFA). The FMS’ convergent and divergent validity, as well as internal consistency, were also tested.

**Results:**

Findings from the EFA and Monte Carlo PCA suggested the retention of three factors (labelled ‘Supportive Management’, ‘Resource Management’ and ‘Time management’). The 3-factor solution explained 51% of the variance in perceived facility management. These results were supported by the results of the CFA (*N* = 381; χ2 = 256.8, df = 61, *p* < 0.001; CFI = 0.94; TLI = 0.92; RMSEA [95% CI] = 0.065 [0.057–0.074]; SRMR = 0.047).

**Conclusion:**

The FMS is an open-access, short, easy-to-administer scale that can be used to assess how health workers perceive facility-level management in LMICs. When used as a regular monitoring tool, the FMS can identify key strengths or challenges pertaining to time, resources, and supportive management functions at the health facility level.

**Supplementary Information:**

The online version contains supplementary material available at 10.1186/s12913-024-10781-y.

## Background

Health governance, leadership, and management are widely recognised as central to health system strengthening efforts and are considered important to ensure the quality and safety of health service delivery, patient satisfaction and increased staff performance and retention [1–5]. This recognition has prompted several calls for research focusing on the nature, quality, and contributions of governance, leadership and management to health systems [6]. Similarly, the World Health Organization (WHO) has identified strengthening governance, leadership, and management as a key strategy to improve health systems within low- and middle-income countries (LMICs) [7], where health systems remain severely under-resourced with regard to management capabilities.

While the terms ‘leadership’ and ‘management’ are often used interchangeably across health policy documents, health systems research, and within health services, others differentiate ‘leadership’– as the act of driving ‘change’ or providing ‘direction, alignment, and commitment’ across multiple actors in a health system–from the concept of ‘management’, which refers to the act of ‘supporting, resourcing, and facilitating day-to-day work’ through the effective mobilisation and utilisation of resources [8–10]. Governance, on the other hand, while also used interchangeably with ‘leadership’ or ‘stewardship’, more broadly refers to the set of *processes*– policies, structures, standards, norms, and practices– that guide decision-making, accountability, and ethical conduct within healthcare organisations, and often implies oversight by boards of directors and regulatory bodies [11]. In this way, management is considered (by some) to be more concerned with operations, general administration, and sufficient availability of both human and material resources. That said, governance, leadership and management are inextricable from quality health service delivery, which, among other factors, depends on leaders with strong managerial skills working to improve performance, accountability and decision-making of health organizations, [12].

While assessing health system governance tends to rely primarily on the use of frameworks [13, 14], several questionnaires and scales have been designed to assess management and leadership [3, 15–19]. The Multi-factor Leadership Questionnaire (MLQ), for example, has long been used as a measure of transformational, transactional, and passive-avoidant forms of leadership [20]. The MLQ, however, is designed to assess leadership *styles*, and therefore does not take into consideration other factors, such as resource and time management, that can also contribute to one’s perception of leadership and managerial support [9]. Similarly, the Leadership Practices Inventory [21], while inclusive of an ‘Observer’ version that allows others to rate the leadership of others, is only available at a fee, thus limiting its accessibility within more resource-constrained contexts. The Foundational Healthcare Leadership Self-assessment (FHLS) [22]; the International Leadership Scale (ILS) [23]; Evidence-Based Practice Nursing Leadership Scale [24] have also all been used within health care settings. A common critique of common management and leadership scales, however, is that like the MLQ and LPI, they were developed based on a Western, individualistic perspective, which may or may not be appropriate to assess leadership across other cultural contexts.

Which form of governance, leadership, and management is preferable in any given context is highly susceptible to socio-political and cultural norms, and speaks to the need to consider ‘the reciprocal influence actors have upon one another’s interests and priorities, and the enabling environment within the health-eco system’ [25]. Whereas governance strengthening efforts tend to take place at a national level, investment in leadership and management for health are frequently concentrated at a district (i.e., within district health management teams; DHMTs) or sub-district level (i.e., within large, district-based, referral hospitals), with the assumption that improvements will ‘trickle-down’ to primary health facilities [26–28]. Consequently, there is a dearth of research focused on *health facility* based leadership and management– despite its proximal influence within primary care settings and decentralized health systems [29]. Better methods to assess, and therefore regularly monitor, facility-level management are therefore necessary if we consider strengthening facility-level management as an equally important element of the governance, leadership, and management health system building block. The specific objective of this study was thus to develop a Facility-level Management Scale (FMS), and to test its reliability among facility-level health workers within LMICs.

## Methods

This study leveraged PERFORM2Scale baseline data, collected through a multi-country survey of health workers. PERFORM2Scale is a multi-country research collaboration whose aim was to strengthen the management of health systems by applying participatory action research within three sub-Saharan African countries [30].

Study participants were comprised of health workers currently employed within a health facility and offering primary health care services. Participants were located across 138 health care facilities, spanning three districts in Uganda (October 2018), three districts in Malawi (November-December 2018), and three districts in Ghana (February-March 2018). All public primary care facilities within the nine selected districts were included across the three countries, except for private and non-governmental organization (NGO) facilities as these do not fall under the full jurisdiction of local governments.

Data was collected in 2018 using a self-administered paper-based questionnaire to health workers. Sample size varied slightly between countries. In Uganda, non-probability convenient sampling techniques were used, whereby all technical health workers that were present at the health facility and/or hospital on the day of data collection were invited to participate in the study. In Malawi, a two-stage probability sampling approach was used, whereby health workers eligible from the district and government facilities were listed for each district and then sampled proportion to the size of each facility’s workforce (*n* = 67). In Ghana, sample size was determined based on published sample size table (Israel 2009), with a precision level of ± 5%, confidence level of 95% and adjusted for non-response rate of 5% resulting a sample size of 252 health workers.

In total, 881 health workers completed an initial, self-report, version of the FMS across Ghana (*n* = 287; 32.6%), Malawi (*n* = 66; 7.5%) and Uganda (*n* = 528; 59.9%) as part of PERFORM2Scale’s baseline survey. Of these, *n* = 119 health workers self-identified as being in a managerial position within their health facility were excluded from the current analysis since the tool was designed to capture health workers perceptions of management. Of the remaining 762 health workers, 69.7% (*n* = 531) were female. The majority were nursing assistants (24.3%), nurses (19.0%), or community health nurses (9.7%). Written consent was obtained from all study participants and all surveys were completed in English as a language commonly spoken among those of high educational achievement across the three countries.

### Development of the initial facility management scale (FMS)

An initial set of nineteen items were developed drawing from extant research on leadership and management for health systems strengthening in LMICs. The original items of the FMS are reported in Table 1. All items were rated using a five-point Likert scale, anchored by Strongly Disagree (= 1) and Strongly Agree (= 5). This original scale was found to have acceptable internal reliability in the current sample (α = 0.88).

**Table 1:** Exploratory factor analysis using principal component factor, showing only factors loadings > 0.40

Item	Factor 1	Factor 2	Factor 3	Factor 4
1. Management supports my daily work efforts	0.544			
2. Problematic personnel are dealt with constructively	0.627			
3. Management makes guidelines available at the health care facility	0.782			
4. Management supports health workers to understand and use clinical guidelines	0.784			
5. Management treats staff in a fair and open manner	0.846			
6. Management effectively resolves conflict between staff	0.713			
7. *Management considers gender issues when addressing staffing at the facility*				
8. Management provides adequate in service training to staff at this facility	0.457			
9. There is a good system for managing shifts so that all staff get a break during working hours			0.811	
10. There is a good system for managing how often staff are on-call at night			0.779	
11. I have regular leave from work			0.642	
12. *My job description corresponds to the reality of my work*				
13. Management supports this facility to maintain equipment and to repair or replace it if is broken		0.566		
14. Management tries hard to avoid/respond to lack of supplies		0.912		
15. Management tries hard to avoid/respond to drug stock-outs		0.933		
16. *Management actively seeks feedback from the community about the quality of care*				
17. Management encourages us to pay attention to differences between women, men, boys and girls when providing health services				0.430
18. Management assures that we are committed to do quality work				0.826
19. Management pays attention that we treat patients with respect and dignity irrespective of their financial status				0.867
Cronbach’s Alpha	0.84	0.76	0.62	0.62

*Note* Factor loadings presented are those of > 0.40 on all factors

### Missing data

The within-item missing data ranged from 2 to 6%. Twelve observations were dropped due to missing data on all the 19 items of the FMS scale. The pattern of missing data was established using Little’s Missing Completely At Random (MCAR) test [31]. The pattern of missing data was not MCAR (*p* < 0.001). The Ordered Logistic model was thus used to impute missing values.

### Data analysis

The construct validity of the FMS was assessed using a hybrid factor analysis approach, as outlined by M Matsunaga [32]. First, cases were randomly assigned to one of two databases for the purposes of examining the underlying factor structure of the data. This resulted in *n* = 375 cases being included in each factor analysis, considered a ‘good’ sample size for the purposes of factor analysis [33]. The first *n* = 375 cases were subjected to Exploratory Factor Analysis (EFA) in order to summarise the data by reducing it to a fewer number of components, or factors [34]. Principal component analysis was carried out to explore the latent structure of the 19-item FMS using an oblique (Promax) rotation. *A priori* criteria for item retention were then applied. First, items were removed where they yielded a factor loadings of < 0.40 (whereby 16% of the variance is explained by the latent variable) [35]. We then identified and removed cross-loading items, or items that loaded considerably on two or more factors. Finally, those items with residual covariances were also removed.

The number of factors retained was determined based on Eigenvalues larger than 1.0, which were further confirmed by running a Monte Carlo PCA [36]. Where the Eigenvalue value generated by the EFA was larger than the criterion value from the Monte Carlo PCA, it was retained. Where the Eigenvalue from EFA was less than the criterion value, it was rejected. Items within each factor were then examined for communalities, prior to labelling each factor according to the set of highly loading components.

To test, or confirm, whether the latent structure of the FMS was an adequate representation of the observed data, the factor structure generated during the EFA was subsequently subjected to Confirmatory Factor Analysis (CFA). Specifically, we fitted the CFA model using maximum likelihood estimation and model fit was established using Root Mean Square Error Approximation (RMSEA), Standardized Root Mean Square Residual (SRMR), Comparative Fit Index (CFI), Tusker-Lewis Index (TLI), and the raw and modified chi-square (χ2) fit statistics [37]. In line with current conventions, CFI and TLI values greater than 0.90 were considered ‘good’ and values greater than 0.95 were considered ‘excellent’; RMSEA and SRMR values of less than 0.05 were considered to reflect excellent model fit, while values more than 0.08 were considered to reflect poor model fit [38, 39]. The same procedures were repeated on the second random sample to confirm construct validity of FMS scale. All factor analyses were conducted using STATA (Version 17). To evaluate the internal reliability of the FMS scale, Cronbach’s alpha was assessed both for each sub-scale and overall scale.

Convergent validity was evaluated by estimating the Average Variance Extracted (AVE) for each construct against its correlation with the other constructs. If the AVE was larger than the construct correlation with other constructs, the convergent validity was considered to be confirmed. Discriminant validity was established where Maximum Shared Variance (MSV) and Average Shared Squared Variance (ASV) were lower than Average Variance Extracted (AVE) for all the constructs.

## Results

### Exploratory factor analysis

The results of the EFA for all 19-items are presented in Table 1.

Overall, three items (*Management considers gender issues when addressing staffing at the facility*; *My job description corresponds to the reality of my work*; and *Management actively seeks feedback from the community about the quality of care*) were found to have a factor loading of less than 0.40 on any given factor and were thus removed from the scale. Finally, the results of the Monte Carlo PCA (see Table 2) suggested the retention of the first three factors, with Eigenvalues of 6.25, 1.39, and 1.29, respectively, resulting in the removal of three additional items (*Management encourages us to pay attention to differences between women, men, boys, and girls when providing health services; Management assures that we are committed to do quality work;* and *Management pays attention that we treat patients with respect and dignity irrespective of their financial status).*

**Table 2:** Results of the monte Carlo PCA, compared to Eigenvalues generated by the exploratory factor analysis

Factor	Eigenvalue	Difference	Proportion	Cumulative	Monte Carlo PCA	Decision
1	6.24723	4.86156	0.3288	0.3288	1.414	Retain
2	1.38567	0.09332	0.0729	0.4017	1.334	Retain
3	1.29235	0.09375	0.068	0.4697	1.275	Retain
4	1.19859	0.20423	0.0631	0.5328	1.225	**Reject**

Ultimately, Factor 1 included items that, when scored highly, describe a supportive style of management, and was thus labeled the ‘Supportive Management’ factor. Factor 2 included items that when scored highly are indicative of effective “resource management”, including the avoidance of stock-outs, and regular repairs of equipment. Finally, Factor 3 included items that, when endorsed, are indicative of supportive time management, including consideration for fair scheduling of duties and annual leave. This was labelled ‘Time Management’. Together, the resulting three-factor solution explained 51% of the variance in perceived facility managerial support.

### Confirmatory factor analysis

**Fig. 1:**
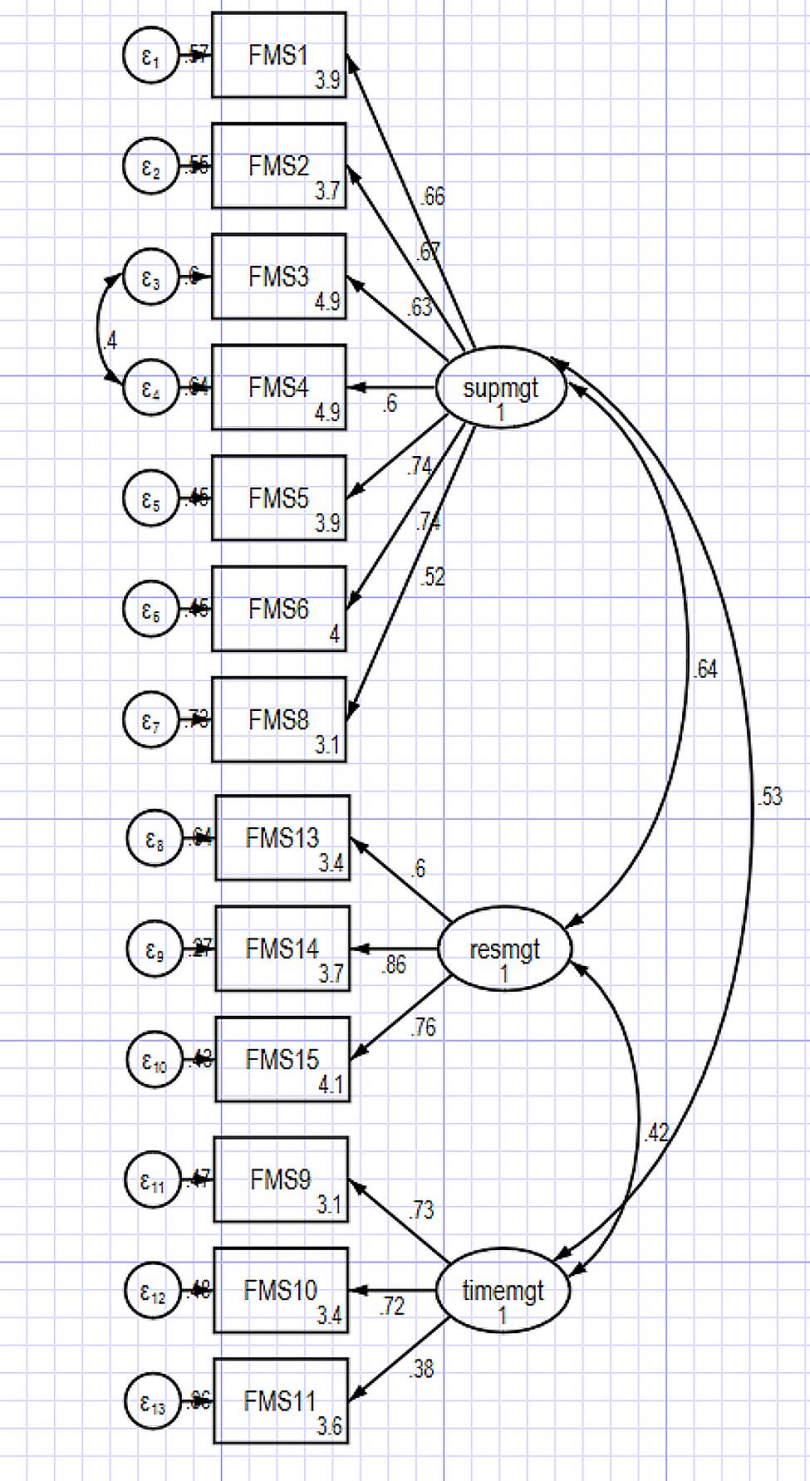
Three-factor correlated structure of the FMS tested using Confirmatory factor analysis. CFA model. *Note* Supmgt = Supportive Management, resmgt = Resource Management, timemgt = Time Management

Results of the CFA on the second half of the data file, carried out with the remaining 13 items (see Fig. 1), yielded good model fit (*N* = 381; χ2 = 256.8, df = 61, *p* < 0.001; CFI = 0.94; TLI = 0.92; RMSEA [95% CI] = 0.065 [0.057–0.074]; SRMR = 0.047). The factor loading for the 13 items were all positive, statistically significant, and of a robust magnitude (Table 3). Table 4 presents the FMS’ inter-factor correlations, which were all significant, moderate, and positive.

**Table 3:** CFA loading’s

Item	Factor	Standardized ($\varvec{\beta }$)	Unstandardized (B)
1. Management supports my daily work efforts	Supportive Management	0.658 (0.024)	1
2. Problematic personnel are dealt with constructively		0.672 (0.024)	1.042 (0.066)
3. Management makes guidelines available at the health care facility		0.629 (0.026)	0.803 (0.054)
4. Management supports health workers to understand and use clinical guidelines		0.597 (0.027)	0.777 (0.056)
5. Management treats staff in a fair and open manner		0.745 (0.021)	1.144 (0.069)
6. Management effectively resolves conflict between staff		0.741 (0.021)	1.137 (0.069)
7. Management provides adequate in service training to staff at this facility		0.523 (0.029)	0.959 (0.076)
8. There is a good system for managing shifts so that all staff get a break during working hours	Time Management	0.726 (0.032)	1
9. There is a good system for managing how often staff are on-call at night		0.720 (0.032)	0.917 (0.076)
10. I have regular leave from work		0.379 (0.038)	0.473 (0.057)
11. Management supports this facility to maintain equipment and to repair or replace it if is broken	Resource Management	0.603 (0.028)	1
12. Management tries hard to avoid/respond to lack of supplies		0.856 (0.019)	1.322 (0.087)
13. Management tries hard to avoid/respond to drug stock-outs		0.755 (0.021)	1.082 (0.073)

*All factor loadings are significant at the *p* < 0.001 level)

**Table 4:** Inter-factor correlations

FMS Sub-Scale	(1)	(2)	(3)
Supportive Management (1)	1		
Resource Management (2)	0.637**	1	
Time Management (3)	0.532**	0.418**	1

**Significant correlation (*p* < 0.01)

### Discriminant and convergent validity

Table 5 presents the Composite Reliability (CR) and Average Variance Extracted (AVE) of the FMS. CR was found to be greater than AVE, in support of the reduced scale’s convergent validity. The AVE of each construct/factor was then compared to the inter-factor correlations and was found to be greater than its correlation with other constructs, in support of the scale’s convergent validity. Discriminant validity was confirmed by AVE > MSV and AVE > ASV.

**Table 5:** Convergent and discriminant validity of the FMS (13 item scale)

Scales dimensions	CR	AVE	AVE^a^	MSV	ASV
Supportive management	0.84	0.429	0.655	0.406	0.344
Resource management	0.78	0.546	0.738	0.406	0.290
Time Management	0.64	0.396	0.629	0.283	0.228

CR = Composite reliability; AVE = Average variance extracted; MSV = Maximum shared variance; ASV = Average shared squared variance

^a^Square root of AVE

## Discussion

Health governance, leadership and management are a key component of any health system and play an important role in strengthening health systems and improving service performance [12, 40]. Within LMICs, health leadership and management strengthening efforts are most often concentrated at district level. Consequently, how health management is experienced or perceived at the level of the health facility is less well understood. Overall, results of the exploratory phase of this study suggest that health-facility-level management is best conceptualised as a three-factor, correlated model characterised by “supportive management”, “time management”, and “resource management”. The emergence of a “supportive management” as a component of health facility management is consistent with more translational approaches focused on building a relationship of trust and confidence between health workers and their managers [41, 42]. Similarly, supportive approaches are consistent with a growing literature which promotes encouraging teamwork and relationships and collective problem solving as key elements of supportive supervision [43–45]. Supportive management thus stands in contrast with more ‘top-down’ (i.e., authoritarian) approaches, or styles based on a system of rewards and punishment (e.g., transactional approaches), which are thought to be less effective for fostering health worker engagement and motivation [12]. Likewise, the emergence of the “time-management” and “resource-management” factors is consistent with the concept of ‘management’ as being more concerned with the day-to-day operations and the sufficient and timely available of human and material resources [8–10]. Taken together, the inclusion of all three factors within the revised version of the FMS is consistent with the idea of ‘leadership’ and ‘management’ as distinct, but related, concepts and that ‘good’ health managers are commonly perceived as those ‘managers who lead’ [46].

Results from the confirmatory phase of the study further suggest that the three-factor structure had acceptable construct, convergent, and discriminant validity, as well as good reliability. Taken together, the development of the FMS, as an easy to use, open-access, short, validated measure of health facility managerial support that can be easily transferred, translated, applied across cadres and within resource constrained settings, and therefore has the potential to improve our understanding and monitoring of staff perceptions of management and leadership at the level of the health facility. Practically, the sub-scales of the FMS can be examined independently, or the FMS items can be totalled as a sum score, whereby higher scores are associated with perceptions of stronger health facility management. Furthermore, the FMS could serve as a useful tool for higher-level management (i.e., district health management teams), helping them detect problematic managerial practices (i.e., around staffing or resource management) and strengthen facility management training programmes, with the ultimate goal to improve staff retention and the quality and safety of service delivery [1–4]. The development of the FMS further addresses calls for more rigorous approaches to scale development within human resource for health programming [47].

Like other measures of managerial practices (e.g., MLQ, LPI), the FMS can be completed by multiple raters, used across cadres, and with multiple subordinates, providing a more reliable view of a manager. The FMS, developed and validated with a sample of healthcare staff across three sub-Saharan African countries, however, further adds to our understanding of how managerial support is perceived within these contexts.

## Limitations

The current study is not without limitations. First, the final FMS may not represent all possible domains of health workers perceptions about leadership and management. Other factors– some of which are measured within some of other scales– are not assessed within the FMS. For example, the ability to delegate, manage change, and create a positive work environment, are not captured by the items contained within the FMS. Second, the public health facilities across the three countries represent varying levels of care and may not be generalizable to all health workers; it is possible that had the scale refinement process been conducted in a different setting, a different set of indicators may have been retained. Thirdly, limited within-country sample sizes, specifically in Malawi, meant we could not ascertain measurement invariance across each context. We therefore strongly encourage that further validation of the FMS take place across other health cadres and across health workers working in both private and not-for-profit health facilities globally.

## Conclusion

In comparison to current tools (e.g., MLQ, LPI), largely developed within Western settings, the FMS allows for the subjective measurement of perceived managerial support, validated across a sample of health workers from three different African countries. Simple and quick to administer, and available open-access (available from Appendix 1 and https://www.perform2scale.org/), the validated FMS has the potential to contribute towards a more accurate understanding of perceptions of managerial support, as a critical determinant of health system performance.

### Electronic supplementary material

Below is the link to the electronic supplementary material.

Supplementary Material 1

## Data Availability

The datasets used and/or analysed during the study are available from the corresponding author on reasonable request.

## References

[1] Nxumalo N, Goudge J, Gilson L, Eyles J (2018). Performance management in times of change: experiences of implementing a performance assessment system in a district in South Africa. Int J Equity Health.

[2] Mannion R, Davies H (2018). Understanding organisational culture for healthcare quality improvement. BMJ.

[3] Mbau R, Gilson L (2018). Influence of organisational culture on the implementation of health sector reforms in low- and middle-income countries: a qualitative interpretive review. Glob Health Action.

[4] Gilson L, Daire J (2011). Leadership and governance within the South African health system. South Afr Health Rev.

[5] Chimwaza W, Chipeta E, Ngwira A, Kamwendo F, Taulo F, Bradley S, McAuliffe E (2014). What makes staff consider leaving the health service in Malawi?. Hum Resour Health.

[6] AHPSR: Alliance for Health Policy and Systems Research (AHPSR). In. Flagship Report 2016 Open mindsets Participatory Leadership for Health Geneva. World Health Organisation; 2016.

[7] Coates MM, Ezzati M, Robles Aguilar G, Kwan GF, Vigo D, Mocumbi AO, Becker AE, Makani J, Hyder AA, Jain Y (2021). Burden of disease among the world’s poorest billion people: an expert-informed secondary analysis of global burden of Disease estimates. PLoS ONE.

[8] Limb M (2016). How does leadership differ from management in medicine?. BMJ.

[9] Waddington C, Egger D, Travis P, Hawken L, Dovlo D. Towards better leadership and management in health: report of an international consultation on strengthening leadership and management in low-income countries, 29 January-1 February, Accra, Ghana. In: Towards better leadership and management in health: report of an international consultation on strengthening leadership and management in low-income countries, 29 January-1 February, Accra, Ghana edn.; 2007.

[10] Reich MR, Javadi D, Ghaffar A (2016). Introduction to the Special Issue on Effective Leadership for Health systems. Health Syst Reform.

[11] Barbazza E, Tello JE (2014). A review of health governance: definitions, dimensions and tools to govern. Health Policy.

[12] Gilson L, Agyepong IA (2018). Strengthening health system leadership for better governance: what does it take?. Health Policy Plann.

[13] Pyone T, Smith H, van den Broek N (2017). Frameworks to assess health systems governance: a systematic review. Health Policy Plan.

[14] Siddiqi S, Masud TI, Nishtar S, Peters DH, Sabri B, Bile KM, Jama MA (2009). Framework for assessing governance of the health system in developing countries: gateway to good governance. Health Policy.

[15] Currie G, Lockett A. Distributing Leadership in Health and Social Care: concertive, conjoint or collective? Int J Manage Reviews 2011, 13.

[16] De Brun A, Rogers L, O’Shea M, McAuliffe E (2019). Understanding the impact of a collective leadership intervention on team working and safety culture in healthcare teams: a realist evaluation protocol. HRB open Res.

[17] Johnson O, Begg K, Kelly AH, Sevdalis N (2020). Interventions to strengthen the leadership capabilities of health professionals in Sub-saharan Africa: a scoping review. Health Policy Plann.

[18] Russell RF, Stone AG (2002). A review of servant leadership attributes: developing a practical model. Leadersh Organ Dev J.

[19] Yukl G (2012). Effective Leadership Behavior: what we know and what questions need more attention. Acad Manage Perspect.

[20] Avolio BJ, Bass BM. Multifactor leadership questionnaire (TM). *Mind Garden, Inc Menlo Park, CA* 2004.

[21] Posner BZ, Kouzes JM (1993). Psychometric properties of the Leadership practices Inventory-updated. Educ Psychol Meas.

[22] Van Hala S, Cochella S, Jaggi R, Frost CJ, Kiraly B, Pohl S, Gren L (2018). Development and validation of the Foundational Healthcare Leadership Self-assessment. Fam Med.

[23] Aarons GA, Ehrhart MG, Torres EM, Finn NK, Roesch SC (2016). Validation of the implementation Leadership Scale (ILS) in Substance use Disorder Treatment organizations. J Subst Abuse Treat.

[24] Shuman CJ, Ehrhart MG, Torres EM, Veliz P, Kath LM, VanAntwerp K, Banaszak-Holl J, Titler MG, Aarons GA (2020). EBP implementation Leadership of Frontline Nurse managers: validation of the implementation Leadership Scale in Acute Care. Worldviews evidence-based Nurs.

[25] World Health Organization.: Open Mindset: Participatory Leadership for Health. In. Geneva: WHO; 2016.

[26] Heerdegen A (2020). Transforming capacity-strengthening in an era of sustainable development. Int J Public Health.

[27] Bonenberger M, Aikins M, Akweongo P, Wyss K (2014). The effects of health worker motivation and job satisfaction on turnover intention in Ghana: a cross-sectional study. Hum Resour Health.

[28] Bossert TJ (2016). Decision space and capacities in the Decentralization of Health Services in Fiji; Comment on Decentralisation of Health Services in Fiji: a decision space analysis. Int J Health Policy Manage.

[29] Ohrling M, Tolf S, Solberg-Carlsson K, Brommels M (2021). Managers do it their way: how managers act in a decentralised healthcare services provider organisation– a mixed methods study. Health Serv Manage Res.

[30] PERFORM2Scale project. In.; Accessed 02 Nov 2021.

[31] Li C (2013). Little’s test of missing completely at Random. Stata J.

[32] Matsunaga M (2010). How to factor-analyze your data right: do’s, don’ts, and how-to’s. Int J Psychol Res.

[33] Comrey AL, Lee HB. A first course in factor analysis. 2nd ed. Psychology; 1992.

[34] Fabrigar LR, Wegener DT, MacCallum RC, Strahan EJ (1999). Evaluating the use of exploratory factor analysis in psychological research. Psychol Methods.

[35] Comrey AL, Lee HB (1992). A first course in factor analysis.

[36] Watkins M. Monte Carlo PCA for Parallel Analysis (Computer Software). *State College, PA: Ed & Psych Associates* 2000.

[37] Hooper D, Coughlan JP, Mullen MR. Structural equation modelling: guidelines for determining model fit. In: *2008*; 2008.

[38] Marsh HW, Hau KT, Wen Z (2004). In search of Golden rules: comment on hypothesis-testing approaches to setting cutoff values for fit indexes and dangers in overgeneralizing Hu and Bentler’s (1999) findings. Struct Equ Model.

[39] Hu L, Bentler PM (1999). Cutoff criteria for fit indices in covariance structure analysis: conventional criteria versus new alternatives. Struct Equ Model.

[40] Mathole T, Lembani M, Jackson D, Zarowsky C, Bijlmakers L, Sanders D (2018). Leadership and the functioning of maternal health services in two rural district hospitals in South Africa. Health Policy Plan.

[41] Aberese-Ako M, Agyepong IA, van Dijk H (2018). Leadership styles in two Ghanaian hospitals in a challenging environment. Health Policy Plann.

[42] Vallières F, Hyland P, McAuliffe E, Mahmud I, Tulloch O, Walker P, Taegtmeyer M. A new tool to measure approaches to supervision from the perspective of community health workers: a prospective, longitudinal, validation study in seven countries. BMC Health Serv Res 2018, 18(1). 10.1186/s12913-018-3595-7 PMC6196473 30348147

[43] Coyle C, Travers Á, Creaner M, Haile-Mariam D, Vallières F: 54supportive supervision for community health workers: a systems-thinking approach. In: Systems thinking for global health: how can systems-thinking contribute to solving key challenges in global health? edn. edited by Vallières F, Mannan H, Kodate N, Larkan F: Oxford University Press; 2022: 0.

[44] Abujaber N, Vallières F, McBride KA, Sheaf G, Blum PT, Wiedemann N, Travers Á (2022). Examining the evidence for best practice guidelines in supportive supervision of lay health care providers in humanitarian emergencies: a systematic scoping review. J Global Health.

[45] Vallières F, Mannan H, Kodate N, Larkan F, McGlacken-Byrne D, Jha S, Abu-Agla A, Badr E, Coyle C, Chikaphupha K et al. Systems Thinking for Global Health: How can systems-thinking contribute to solving key challenges in Global Health? 2022.

[46] Galer JB, Buxbaum AS, Vriesendorp S, Peza LDl, Ellis A, Dwyer J, Hall MJ, Huber SC, Miller J, Johnson S et al. Managers who lead: a handbook for improving health services. In: *2005*; 2005.

[47] Borghi J, Lohmann J, Dale E, Meheus F, Goudge J, Oboirien K, Kuwawenaruwa A et al. How to do (or not to do)… measuring health worker motivation in surveys in low- and middle-income countries. Health policy and planning. 2018;33(2):192–203. 10.1093/heapol/czx153 PMC5886192 29165641

