# Supplementary material for: A new scale to assess health-facility level management: the development and validation of the facility management scale in Ghana, Uganda, and Malawi
Source: BMC Health Serv Res. 2024 Mar 25;24:371. doi: 10.1186/s12913-024-10781-y (PMC10964570; doi:10.1186/s12913-024-10781-y)
Supplement: Supplementary file 1 — Supplementary Material 1 [file 12913_2024_10781_MOESM1_ESM.docx]

**Appendix 1 – the Facility Management Scale (Version 1.0)**

*Please indicate the extent to which you agree or disagree with the following statements in regards to management at your health facility. Please answer every question by ticking the appropriate box, not leaving any blank.*

|  | **Strongly Disagree** | **Disagree** | **Neither Agree nor Disagree** | **Agree** | | **Strongly Agree** | **Not Applicable** |
| --- | --- | --- | --- | --- | --- | --- | --- |
|  | **1** | **2** | **3** | **4** | | **5** | **6** |
| 1. Management supports my daily work efforts | ☐ | ☐ | ☐ | ☐ | | ☐ | ☐ |
| 1. Problematic personnel are dealt with constructively | ☐ | ☐ | ☐ | ☐ | | ☐ | ☐ |
| 1. Management makes guidelines available at the health care facility | ☐ | ☐ | ☐ | ☐ | | ☐ | ☐ |
| 1. Management supports health workers to understand and use clinical guidelines | ☐ | ☐ | ☐ | ☐ | | ☐ | ☐ |
| 1. Management treats staff in a fair and open manner | ☐ | ☐ | ☐ | ☐ | | ☐ | ☐ |
| 1. Management effectively resolves conflict between staff | ☐ | ☐ | ☐ | ☐ | | ☐ | ☐ |
| 1. Management provides adequate in service training to staff at this facility | ☐ | ☐ | ☐ | ☐ | | ☐ | ☐ |
| 1. There is a good system for managing shifts so that all staff get a break during working hours | ☐ | ☐ | ☐ | ☐ | | ☐ | ☐ |
| 1. There is a good system for managing how often staff are on-call at night | ☐ | ☐ | ☐ | ☐ | | ☐ | ☐ |
| 1. I have regular leave from work | ☐ | ☐ | ☐ | ☐ | | ☐ | ☐ |
| 1. Management supports this facility to maintain equipment and to repair or replace it if is broken | ☐ | ☐ | ☐ | ☐ | | ☐ | ☐ |
| 1. Management tries hard to avoid/respond to lack of supplies | ☐ | ☐ | ☐ | ☐ | | ☐ | ☐ |
| 1. Management tries hard to avoid/respond to drug stock-outs | ☐ | ☐ | ☐ | | ☐ | ☐ | ☐ |
